# Aggression inoculates against PTSD symptom severity—insights from armed groups in the eastern DR Congo

**DOI:** 10.3402/ejpt.v4i0.20070

**Published:** 2013-05-13

**Authors:** Tobias Hecker, Katharin Hermenau, Anna Maedl, Maggie Schauer, Thomas Elbert

**Affiliations:** 1 Department of Psychology, University of Konstanz, Konstanz, Germany; 2 vivo international, Allensbach, Germany

**Keywords:** Appetitive aggression, PTSD, defense, building block effect, excombatants, DR Congo

## Abstract

**Background:**

In the ongoing conflict in the Democratic Republic of the Congo (DRC), combatants are exposed to massive forms of violence and other traumatic stressors. Nevertheless, many combatants do not suffer from trauma-related disorders, although they have experienced numerous traumatizing events. Perceiving aggressive behavior as fascinating and arousing might be a defense against trauma-related disorders in the violent environment of war and conflict.

**Objective:**

Thus, in this study we investigated the relationship between the exposure to traumatic stressors, appetitive aggression, and posttraumatic stress disorder (PTSD) symptom severity. We hypothesized that cumulative traumatic experiences correlated positively and appetitive aggression negatively with PTSD symptom severity.

**Method:**

In total, 105 voluntary male combatants from different armed groups in the eastern DRC took part in this study. In a semistructured interview, respondents were questioned about their exposure to traumatic stressors, the extent of appetitive aggression (Appetitive Aggression Scale) and their PTSD symptom severity (PTSD Symptom Scale - Interview).

**Results:**

A multiple sequential regression analysis showed that traumatic events were positively related to PTSD symptom severity. For participants with low to medium PTSD symptom severity, appetitive aggression correlated negatively with PTSD symptom severity.

**Conclusions:**

The results of this study provide further support for earlier findings that repeated exposure to traumatic stressors cumulatively heightens the risk of PTSD and revealed that appetitive aggression buffers the risk of developing PTSD symptoms under certain circumstances. Thus, the perception of aggressive behavior as fascinating and arousing seem to help combatants to adapt to violent environments but may also be one reason for recurrent failure of reintegration programs for excombatants.

The eastern Democratic Republic of the Congo (DRC) has been trapped in ongoing cycles of war and violence for more than two decades, with thousands of soldiers and combatants serving in numerous local militias and armed groups (Davis & Hayner, 2009; Romkema, 2007). Combatants are exposed to war and violence on a daily basis. The brain adapts to frequent stressors and danger, such as those posed by the conditions in the eastern DRC, by prioritizing a stress-responsive pathway. This pathway helps the individual to react to danger with aggression or flight, but it is also related with a higher risk of mental illness (Elbert, Rockstroh, Kolassa, Schauer, & Neuner, 2006). Exposure to severe and traumatic stress may lead to the development of posttraumatic stress disorder (PTSD). Prior research has consistently shown that the greater the cumulative exposure to traumatic experiences, including organized violence, the greater the risk of trauma-related disorders, including depression or substance abuse (Amone-P'Olak, Garnefski, & Kraaij, 2007; Catani, Jacob, Schauer, Kohila, & Neuner, 2008; Chapman et al., 2004; Edwards, Holden, Anda, & Felitti, 2003; Hermenau et al., 2011).

In a study with child soldiers from Uganda and the DRC, however, Bayer, Klasen, and Adam (2007) found that only 35% of the former child soldiers suffered from PTSD, despite having experienced a high number of traumatic events. Beyond that, Pfeiffer and Elbert (2011) reported a negative relationship between the time spent with the rebels and PTSD symptom severity. They concluded that many combatants had adapted to the violent circumstances of an armed group. Elbert, Weierstall, and Schauer (2010) argued that the combatant's partial defense against trauma-related disorders, in spite of having experienced tremendously distressing events and ongoing threats of war and death, might be best explained in terms of the phenomenon of “appetitive aggression”. Aggression is often categorized as either a reactive self-defense response or alternatively as a behavior carried out for some instrumental gain (Anderson & Bushman, 2002). In contrast, appetitive aggression attempts to capture a rather hedonic form of aggressive behavior that we frequently observe in the field. Appetitive aggression is thus conceptualized as perceiving aggressive behavior toward others as fascinating, arousing, and thrilling (Hecker, Hermenau, Maedl, Elbert, & Schauer, 2012). Former child soldiers and excombatants report that their experience of war brought about a gradual transformation in their perception of violence: at first it was frightening, but with repeated experience it became not only normal and acceptable, but even exciting and arousing (Maclure & Denov, 2006). How can we explain this change in people's response to violence? In a peaceful society, moral standards, social customs, and laws inhibit extreme forms of violence including killing of humans (Elbert et al., 2010). Life in an extremely violent environment often breaks these socially learned inhibitions and moral standards (Engen, 2008). This can, for example, be achieved through dehumanization of the enemy (Staub, 2006) and initiation rites in armed groups, including killing of relatives (Amone-P'Olak, 2004). Elbert et al. (2010) developed this idea further by arguing that in analogy to the fear network in PTSD, an associative interconnected set of representations (sensory, cognitive, and affective) related to the fearful experiences, perpetrators form a hunting network, marked by approach rather than avoidance to violence cues. Whereas exposure to violent acts leads to an extension of the fear network, arousing or appetitive elements that arise during the perpetration of violence are integrated into the hunting network. In contrast to violent behavior of psychopathy or sociopathy, which is seen as emotionally “cold” and driven purely by instrumental motives, combatants report perpetrating violence as highly arousing (Weierstall & Elbert, 2012). Furthermore, we view appetitive aggression not as pathological *per se* but as an adaptation in a very bloody, cruel, and violent environment to overcome the learned inhibition of killing other humans. Seen in terms of the approach/avoidance dichotomy of emotion put forward by Lang, Bradley, and Cuthbert (1998), appetitive aggression and its attendant hunting network can be viewed as an approach behavior, whereas fearful responses that generate a fear network can be seen as an avoidance behavior (Weierstall & Elbert, 2012).

In a study in the DRC, former combatants reporting high levels of appetitive aggression were more likely to volunteer for duty and to join as minors. They also reported a higher number of perpetrated violence types (Hecker et al., 2012). For child soldiers, perceiving the perpetration of violence as fascinating and arousing seems to be adaptive for survival in a violent environment. Being more appetitively aggressive is advantageous in this extremely violent environment, with people high in this factor occupying higher positions in armed groups, as well as being both acknowledged and feared by peers (Crombach, Weierstall, Hecker, Schalinski, & Elbert, in press; Hermenau, Hecker, Maedl, Schauer, & Elbert, 2013).

Furthermore, we found that perpetrating violence is perceived as traumatic for some combatants, but not for all. Therefore, it is important to take the combatant's perception of perpetrating violence into account (Hecker et al., 2013). Some combatants consider perpetrating violence to be fascinating and arousing, with voluntary combatants seeming to suffer less from the consequences of perpetrating violence. Elbert et al. (2010) suggested that the appetitive and fascinating element of violence prevents the incorporation of the cruel, genuinely traumatizing experiences into the fear network (Elbert et al., 2006; Elbert & Schauer, 2002). Therefore, we argue that combatants who perceive perpetrating violence to be fascinating and arousing are more likely to integrate sensory input, cognitions, and emotions that are linked to the perpetration of violence into the hunting network, whereas perceiving the perpetration of violence as frightening and disgusting leads to an extension of the fear network. In concordance with this, two studies have found a negative relationship between PTSD symptom severity and appetitive aggression, one in Ugandan child soldiers (Weierstall, Schalinski, Crombach, Hecker, & Elbert, 2012) and the other in Rwandan genocide perpetrators (Weierstall, Schaal, Schalinski, Dusingizemungu, & Elbert, 2011). Consequently, Elbert et al. (2010) concluded that appetitive aggression buffers the risk of PTSD as the integration of violent cues into the hunting network (and not into the fear network) may reduce the likelihood of a trigger-related activation of the fear network. On the other hand, they argue that this protective effect may wane if the combatant exceeds a certain level of traumatization due to an overlap of the hunting and the fear network. As the number of items that are linked to the fear network increases, the higher the likelihood of the fear network being triggered. Consequently, the combatant experiences trauma-related symptoms.

## Objective

In this study, we investigated the relationship between appetitive aggression, exposure to traumatic experiences, and PTSD symptom severity in former combatants in the eastern DRC. In this conflict, spanning more than two decades, many young men volunteer for duty in one of several armed groups. In some areas of North Kivu up to 70% of the young men have been associated with armed groups (Coalition to Stop the Use of Child Soldiers, 2010; Romkema, 2007). To achieve the successful demobilization and integration of former combatants, it is necessary to establish and test models that predict how the life in violent environments shapes young men, as well as why some suffer severely from psychological dysfunction and mental disorders while others adapt well to this harsh environment.

In recent reports on data from the same population, we found that appetitive aggression is linked to joining an armed group voluntarily (Hecker et al., 2012). Furthermore, perpetrated violence was not correlated with PTSD symptom severity in voluntary combatants and they perceived perpetrating violence as more fascinating and arousing than forcibly recruited combatants (Hecker et al., 2013). Hence, we concluded that forcibly recruited and voluntary combatants process violent cues differently. Whereas forcibly recruited combatants are more likely to integrate violent cues into their fear network, voluntary combatants may integrate violent cues in the hunting network, which Elbert et al. (2010) view as the underlying mechanism of the buffering effect of appetitive aggression. In this study, we therefore examined the voluntary combatants (for further details about differences between forcibly recruited and voluntary combatants see Hecker et al., 2013).

We hypothesized that: (1) according to the dose-effect or building block effect (Anda et al., 2006; Mollica, Poole, & Tor, 1998; Neuner et al., 2004) traumatic event types correlated positively with PTSD symptom severity; and (2) we predicted a protective influence of appetitive aggression on PTSD symptom severity. Consequently, we hypothesized a negative correlation between appetitive aggression and PTSD symptom severity. However, according to Elbert et al. (2010), the negative relationship between appetitive aggression and PTSD symptom severity may wane if the level of traumatization exceeds a certain threshold. This is plausible, as a ceiling effect of symptom severity is inherent in every diagnostic instrument. Following Weierstall, Bueno Castellanos, Elbert and Neuner (2013) we thus hypothesized: (3) we detect the protective influence of appetitive aggression on PTSD symptom severity if we exclude combatants with the highest level of traumatization from our analyses.

## Method

### Participants

Between March and May 2011, 224 face-to-face interviews were conducted in Goma in the province of North Kivu, DRC. From this sample, 107 combatants reported that they volunteered to join an armed group. Two interviews were excluded from all analyses because for logistical reasons, they could not be completed. Most interviews, 60% (*n*=63), took place at a UN demobilization transit camp, 38% (*n*=40) were conducted at a reintegration center for former child soldiers and former combatants and 2% (*n*=2) at a military detention facility. As is typical for “new wars” (Elbert et al., 2006; Shaw, 2000), objective boundaries between forcible and voluntary recruitment are often difficult to draw. This held true in the conflict in the eastern DRC. In specific cases where it was unclear from their history, we relied on the individual combatant's subjective perception of his recruitment.

All participants were male (*N*=105). The mean age was 25.72 (SD=7.45, range: 15–50). In total, 69% (*n*=72) were born in the DRC and 31% (*n*=33) in Rwanda. Participants belonged to a variety of armed groups and forces, including “Forces Démocratiques pour la Libération du Rwanda (FDLR)”, “Congrés National pour la Défense du Peuple (CNDP)”, several local “Mai Mai” groups and the Congolese Government Army (FARDC). On average, they served as combatants for 8.02 years (SD=5.75, range: 0.44–28). The mean age, with which they joined an armed group for the first time, was 16.73 years (SD=6.29, range: 0–31). In total, 21% (*n*=22) fulfilled the clinical diagnosis of PTSD. The time since the participants demobilized ranged from 1 week to 6 years. The great majority (75%) of the participant left their armed group within the year before the assessment. Less than 10% of the participants demobilized more than 3 years prior to the assessment.

### Procedure

Four clinical psychologists (T. H., K. H., A. M., and M. S.) and a nurse trained in psychological assessment from the University of Konstanz, who all had extensive work experience in Africa's conflict zones, conducted the semistructured interviews with the help of three interpreters. The interpreters were trained in the concepts of mental disorders and aggression before the assessment. All instruments were translated into Kiswahili, Kinyarwanda, or Lingala, and the translation was intensely discussed to guarantee a precise interpretation. Two of the experts could understand the native languages well enough to continuously supervise and assure valid translation. The interviewers further standardized the form of assessment by practicing in joint interviews to achieve a high inter-rater reliability. Subsequently, one interviewer and one translator individually interviewed each interviewee in a calm and quiet setting. Each interview took on average one and a half hours.

The Ethical Review Board of the University of Konstanz, the United Nations’ mission in the DRC (MONUSCO) and the respective Congolese non-governmental non-profit organization in charge of the reintegration center approved this study. Due to the participants’ illiteracy, we collected oral informed consent. In addition, we asked the respective institutions for permission to interview underage child soldiers, as their caregivers were either dead or not available. Participants received financial compensation of about $2 US. Other aspects of the data gathered during the extensive investigations are presented by Hecker et al. (2012, 2013) and Hermenau et al. (2013).

### Measures

After an informal conversation and the informed consent, the first part of the interview consisted of sociodemographic information (e.g., place and year of birth). Then, the former combatants were interviewed about their military career (e.g., how they were recruited and length of service).

Exposure to traumatic life experiences over the combatant's entire lifetime was assessed using a checklist of 20 war—and non-war-related potentially self-experienced and witnessed traumatic events (e.g., physical assault, assault by weapon, rape, life-threatening injury, accident). This checklist was a version of a previously published checklist (Neuner et al., 2004) that we adapted to fit the cultural context. This checklist showed a high test–retest reliability (*r*=0.73, *p*<0.001) and significant accordance with the CIDI Event List (Ertl et al., 2010) in an earlier study with former child soldiers in the Great Lakes Region. The number of times a specific event had been experienced was not assessed, as distorted memory in PTSD renders this measure unreliable (Elbert & Schauer, 2002; Kolassa & Elbert, 2007; McNally, 2006). For the analyses, we computed a sum score of all traumatic event types (range: 0–20). On average, the participants reported *M*=13.91 (SD=2.56, range: 8–19) different traumatic event types.

The PTSD symptom severity was determined by using the PTSD Symptom Scale - Interview (PSS-I; Foa, Riggs, Dancu, & Rothbaum, 1993). The PSS-I assesses the 17 DSM-IV symptom criteria for PTSD on the basis of the subjectively most traumatic event that occurred at least 1 month prior to the assessment. All items refer to symptoms experienced in the previous month. Each of the items was answered on a 4-point scale ranging from not at all/only one time (0) to five or more times per week/almost always (3). The PSS-I is widely used to diagnose PTSD and has good psychometric properties (e.g., Cronbach's *α*=0.86 and inter-rater reliability=0.93; Foa et al., 1993; Foa & Tolin, 2000). It has been validated for various cultural settings and also for former child soldiers (Ertl et al., 2010). We analyzed the data dimensionally so that potential cultural differences could be taken into account. Therefore, we computed a PTSD severity score (range: 0–51) by adding all symptom scores. On average, the participants reported a PTSD severity score of *M*=9.14 (SD=8.57, range: 0–45). In this sample, the Cronbach's alpha coefficient was 0.90.

Appetitive aggression was assessed with the Appetitive Aggression Scale (AAS; Weierstall & Elbert, 2011), which has been validated with more than 1,600 excombatants and proven its good psychometric properties in comparable samples in Uganda (Weierstall et al., 2012), Rwanda (Weierstall et al., 2011), and the DRC (Hecker et al., 2012). The AAS consists of 15 items regarding the perception of violence or appetitive aggression (e.g., “Is it exciting for you if you make an opponent really suffer?”, “Once fighting has started do you get carried away by the violence?” or “Is fighting the only thing you want to do in life?”). The interviewer rated the level of the interviewee's agreement on a 5-point Likert scale ranging from disagree (0) to agree (4). The items were based on the definition of the instrumental aggression subtype according to Vitiello and Stoff (1997) and the ICD-10 addiction criteria (World Health Organization, 1992). Further items were compiled on the basis of interviews with child soldiers about the appetitive experience of aggression and violence (Elbert et al., 2010). In the validation study, the AAS score showed a Cronbach's alpha coefficient of 0.85 and further analyses revealed that the AAS measures a distinct construct of human aggression (for further details see Weierstall & Elbert, 2011). For the analysis, we computed a sum score of all 15 items. It ranged from 0 to 60. On average, the participants reported an AAS score of *M*=27.69 (SD=13.70, range: 1–60). In the present sample, the Cronbach's alpha coefficient was 0.88. In a multiple regression analysis of the same sample (see also Hecker et al., 2012), which explained 26% of the variances of the AAS score, the AAS score correlated significantly with perpetrated violence types (β=0.40), voluntary enlistment (β=0.20) and joining as a child (β=0.14).

### Data analysis

All analyses used a two-tailed *α*=0.05. First, we conducted a multiple sequential regression analysis including all voluntary combatants (*N*=105) to investigate the correlation between PSS-I score, the number of traumatic event types, and the AAS score. In the first step, only the number of traumatic event types was included as a predictor. In the second step, the AAS score was added to the model. The regression model fulfilled all necessary quality criteria for linear regression analyses. The residuals did not deviate significantly from normality (Kolmogorow-Smirnov-*Z*=1.14, *p*=0.150), linearity, or homoscedasticity. No univariate outliers could be identified. However, Cook's distance revealed four multivariate outliers (2 *SD*>*M*). Consequently, these multivariate outliers were excluded from the analysis. The maximum variance inflation factor did not exceed 1.12. Hence, we do not need to take multicollinearity into account.

In a second step, we excluded all combatants with a PSS-I score higher than 21 (Weierstall et al., 2013). These were the upper 10% (*n*=9) of this sample. Then, we conducted again a multiple sequential regression analysis as we did earlier. Again, the regression model fulfilled all necessary quality criteria for linear regression analyses. The residuals did not deviate significantly from normality (*Kolmogorow-Smirnov-Z*=1.11, *p*=0.174), linearity, or homoscedasticity. No univariate outliers could be identified. However, Cook's distance revealed five multivariate outliers (2 *SD*>*M*). As a result, these multivariate outliers were excluded from the analysis. The maximum variance inflation factor did not exceed 1.13. Hence, we do not need to take multicollinearity into account.

## Results

The first regression model with only the number of traumatic event types as a predictor of the PTSD symptom severity score explained 7% of the variability of the PSS-I score (*R* ^*2*^=0.08, *F*(1, 99)=8.27, *p*=0.005). As shown in Table 1, the number of traumatic events correlated positively with the PSS-I score. Adding the AAS score as an additional predictor did not improve the model (Δ*R* ^2^<0.01, *F*(1, 98)=0.70, *p*=0.405). In other words, our analysis revealed that only the number of traumatic event types correlated significantly with the PTSD symptom severity and not the AAS score. Thus, we replicated the building block effect of cumulative exposure to traumatic stressors but did not find a protective influence of appetitive aggression on PTSD symptom severity for all combatants.

**Table 1:** Results of regression analysis predicting PSS-I

	PSS-I score			
	
Predictor variables	*B*	SE of *B*	β	*T*
Step 1				
Traumatic event types	0.89	0.27	0.32	3.31***
				
Step 2				
Traumatic event types	1.02	0.29	0.36	3.57***
AAS Score	−0.75	0.06	-0.13	-1.31

Notes: *R* ^2^=0.08, *N*=101, *B*, unstandardized regression weight; SE, standard error; β, standardized regression weight; *T*, *t*-test statistics;

*** *p*≤0.001.

### Exclusion of the combatants with a high PTSD symptom severity

**Fig. 1:**
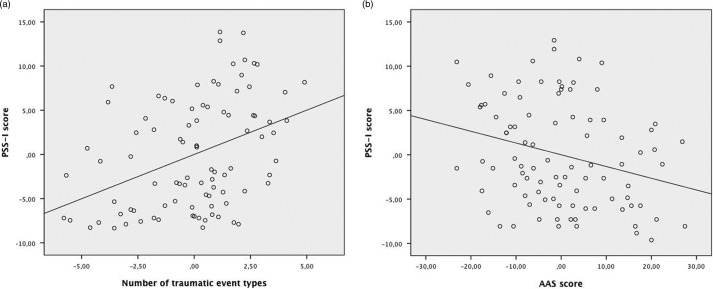
(a) Partial regression from the number of traumatic event types to the PSS-I score (building block effect). (b) Partial regression from the AAS score to the PSS-I score (protective influence).

Given a ceiling effect for trauma symptom severity, we excluded all combatants with a PSS-I score higher than 21. The regression model with only the number of traumatic event types as a predictor of the PTSD symptom severity explained 11% of the variability of the PSS-I score (*R* ^2^=0.12, *F*(1, 89)=12.49, *p*=0.001). After adding the AAS score as an additional predictor, the regression model explained 17% of the variance. The change in *R* ^2^ was significant (Δ*R* ^2^=0.06, *F*(1, 88)=6.61, *p*=0.012). As shown in Table 2, the number of traumatic event types was positively related to the PSS-I score. The AAS score correlated negatively with the PSS-I score. In other words, after excluding the combatants with a PSS-I score higher than 21, we found the building block effect and we also noticed a protective influence of appetitive aggression on the PTSD symptom severity (see Fig. 1).

**Table 2:** Results of regression analysis predicting PSS-I score (excluding combatants with a PSS-I score higher than 21)

	PSS-I score			
	
Predictor variables	*B*	SE of *B*	β	*T*
Step 1				
Traumatic event types	0.86	0.25	0.35	3.53***
Step 2				
Traumatic event types	1.08	0.25	0.44	4.29***
AAS Score	−0.13	0.05	−0.26	−2.57*

*Notes: R* ^2^=0.18, *N*=91; *B*, unstandardized regression weight; SE, standard error; β, standardized regression weight; *T*, *t*-test statistics;

* *p*≤0.05

*** *p*≤0.001.

## Discussion

The results of this study revealed that the number of traumatic event types correlated positively with PTSD symptom severity. Concordant with prior research (Mollica et al., 1998; Neuner et al., 2004), our data thus confirmed the building block effect in former combatants: repeated exposure to different types of traumatic stressors cumulatively heightens the risk for PTSD symptoms. On average, the former combatants reported that they had experienced about 14 different types of traumatic stressors. Following Neuner et al. (2004), for a sample of civilian survivors we would expect PTSD rates between 50 and 60%. However, only 20% of the present sample fulfilled the clinical criteria for a PTSD diagnosis. Additionally, the former combatants reported on average only a low PTSD symptom severity. Elbert et al. (2010) suggested that appetitive aggression might explain why many combatants do not suffer from trauma-related disorders, even if they had gone through tremendously distressing experiences and ongoing threats of war and death. They suggested that the appetitive and fascinating element of violence prevents the incorporation of the cruel, genuinely traumatizing experiences into the fear network (Elbert et al., 2006). In agreement with prior findings (Weierstall et al., 2011, 2012), we found a negative correlation between appetitive aggression and PTSD symptom severity in this sample, i.e., replicated a protective influence of appetitive aggression. However, consistent with Weierstall et al. (2013) we found this protective influence only after excluding those combatants showing the highest PTSD symptom severity. In concordance, Elbert et al. (2010) argued that there is no ultimate defense against trauma-related disorders and the protective influence of appetitive aggression on PTSD symptom severity may wane as the level of traumatization exceeds a certain threshold. They argue, that due to recurrent traumatization, the fear network is extended and may overlap with the hunting network. Consequently, positive or appetitive memories that are linked to the perpetration of violence may not only trigger the hunting network but also the fear network.

In line with Elbert et al. (2010) and our prior findings (Hecker et al., 2012, 2013; Hermenau et al., 2013), we argue that perceiving aggressive behavior as fascinating and arousing can be advantageous in a cruel and violent environment such as that experienced in an armed group, as it reduces the risk of trauma-related disorders such as PTSD.

On the one hand, appetitively aggressive combatants seem to be less likely to perceive perpetrating violence as traumatic (Hecker et al., 2013). On the other hand, they seem to be better prepared to cope with traumatic life experiences up to a certain level. Hence, appetitively aggressive combatants have a higher chance of survival in violent environments but may have difficulties integrating into civil society after demobilization. However, further research is needed to investigate how appetitive aggression develops and how it serves as a buffer against the risk of developing PTSD symptoms. The protective influence of appetitive aggression on PTSD symptom severity has been shown in different conflict zones in Africa and South America and seems to be robust. Future studies should focus on possible moderating or mediating effects of appetitive aggression. For example, it may moderate or mediate the building block effect. If a combatant experiences a great variety of traumatic stressors too frequently, the building block effect may outweigh the protective influence of appetitive aggression. Future research might reveal whether there is a general or an individual threshold, depending on other factors, such as the meaning of the events and the conflict at large for the individual (Hecker et al., 2013; Schauer, Neuner, & Elbert, 2011). Furthermore, future studies may focus on alcohol and substance abuse and its interrelation to perpetrated violence and appetitive aggression as well as factors that help to overcome the threshold for killing another human, e.g., dehumanization of the enemy or in-group and out-group perception or dissociative experiences.

The degree to which the results of the present study can be generalized is limited. First, the cross-sectional study design and the specific sample would not allow us to establish causality. Although we interviewed all voluntary excombatants who were enrolled in the demobilization program of the United Nations and the reintegration program of the Congolese organization in charge of the reintegration center at the time of assessment, this sample may not necessarily be representative for voluntary excombatants. Moreover, the sample consists mainly of deserted combatants, who might not be comparable to active combatants. Although many deserted combatants reported appetitive aggression, difficult living conditions (e.g., lack of weapons, medical supply, food) and traumatic experiences (e.g., life danger, severe injuries or witnessing killing of comrades) in the armed groups made them leave the armed group. We would expect higher appetitive aggression and therefore a better psychological functioning among active combatants.

In this study, we only included combatants who had voluntarily joined an armed group. Although we tried to rate the combatant's own perception of his recruitment, in some cases the decision to join was the result of limited choices in a resource-poor region of instability (Guy, 2009; Schauer & Elbert, 2010). This sometimes blurred the boundaries between forcible and voluntary recruitment and might have influenced the results of the present study. The excombatants talked openly about appetitive aggression. Generally, the respondents greatly appreciated the opportunity to detail their view of the situation in the eastern DRC and to report their own experiences. A potential bias, like the influence of social desirability or of demand characteristics, can never be ruled out for subjective reports. However, the validity of the reported number of different types of traumatic life experiences has been assured by a positive association between hair cortisol levels and the number of lifetime traumatic event types in former child soldiers in an earlier study (Steudte et al., 2011).

## Conclusions

This study has added further support to the notion that repeated exposure to different types of traumatic stressors cumulatively heightens the risk of trauma-related suffering in former combatants. With this study, we validated prior findings from other conflict zones in Africa and Latin-America (Weierstall et al., 2011, 2012; 2013) showing that appetitive aggression may provide a defense against trauma-related disorders under certain circumstances. Our findings indicate that some former combatants seem to adapt to the cruel and violent environment of armed groups and are able to cope with the adverse conditions. After demobilization, attraction to violence and aggressive behavior, however, pose challenges for the integration of excombatants into civil society as it heightens the risk of voluntary rerecruitment (Hermenau et al., 2013). Thus, our findings may offer one possible explanation at the individual level as to why struggles to end the ongoing conflict in the DRC have engendered so little success. To address the combatants’ needs and to improve reintegration programs, we advocate adding mental health components to reintegration programs. In addition to trauma-related sufferings, attraction to violence and enhanced levels of aggressive behavior must be addressed. Demobilization and reintegration of former combatants remain important for solving ongoing conflicts and re-establishing a legitimate authority that provides the public services needed for the development of a peaceful society.
